# Green synthesize of copper nanoparticles on the cotton fabric as a self-regenerating and high-efficient plasmonic solar evaporator

**DOI:** 10.1038/s41598-023-40060-5

**Published:** 2023-08-07

**Authors:** Maedeh Simayee, Azam Iraji zad, Ali Esfandiar

**Affiliations:** 1 Center for Nanoscience and Nanotechnology, Institute for Convergence Science and Technology (ICST), Sharif University of Technology, Tehran, Iran; 2 Department of Physics, Sharif University of Technology, Tehran, Iran

**Keywords:** Environmental sciences, Materials science

## Abstract

Harvesting solar energy, as a clean and abundant resource, in the photothermal process, is the winning point of solar steam generation (SSG) systems. Herein, copper plasmonic nanoparticles were synthesized through a green method via red sanders extraction on the cotton fabric as the reducing matrix. The prepared fabrics were analyzed using FESEM, EDS, XRD, PL, Raman, and contact angle. The treated fabric on the stitched PU foam with cotton yarns with bio-inspired jellyfish structure was used for heat localization and water transmission, simultaneously. The evaporation rate, enhancement, and conversion efficiency of the plasmonic SSG were 1.73 kg m^−2^ h^−1^, 179%, and ~ 98%, under one sun irradiation, respectively. The quality of the collected water was investigated via induced coupled plasma which presents the proper solar desalination (> 99.83% for filtration of Na^+^ ion). Regenerating features of the treated fabric along with the simple and cost-effective preparation method promises viable aspects of our system for large-scale applications.

## Introduction

One of the most important and current crises for all communities is potable water preparation^1^. Seawater desalination is a practical solution to address this demand through three traditional methods including multiple-effect, multiple-stage flash, and reverse osmosis desalination^2^. The energy consumption of these mature technologies is mostly supplied by fossil fuels leading to opposing ecological impacts that make them non-sustainable^3^. On the contrary, interfacial solar steam generation (ISSG) systems by harvesting the inexhaustible^4^, free, clean, and renewable^1^ solar energy is an alternative and feasible attitude^5^.

In an ISSG system, solar irradiation traps the solar absorber floating on the water's surface. Sunlight absorption in a solar absorber occurs through a photothermal material to induce a high surface temperature promoting vaporization^6^. Different kinds of photothermal materials (e.g. plasmonic metals^7, 8^, semiconductors^9^, and carbon-based materials^10^ for heat localization were employed as the sunlight absorber in solar-based evaporators^11–13^.

The light absorption process in metallic materials is followed by the excitation of the conduction band electrons to higher energy states through interband transitions^14, 15^. In some metallic nanoparticles, so-called plasmonic metals, the localized surface plasmon resonance (LSPR) effect is the responsible photothermal mechanism. By frequency matching of the natural metal surface electrons oscillations with the irradiated photons, a resonant photon-induced coherent oscillation of charges occurs known as the LSPR effect^16^. Near-field enhancement, hot electron generation, and photothermal conversion are three induced phenomena that arise sequentially in the mentioned effect^17^. The illumination of plasmonic nanoparticles under their resonance wavelengths leads to the oscillation of the electron gas^18^. Hot electrons as a result of the followed-electron excitation from the occupied to unoccupied states decay through radiative emission and electron–electron scattering^19^. In the last decadence process, redistribution of the hot electron energy causes a fast increase in the localized surface temperature^20^. This temperature increment is followed by equilibrium cooling leading to an energy transfer from the hot electrons to the lattice through electron–phonon and phonon–phonon interactions^21^.

Besides gold and silver, as the most common plasmonic metals, some others such as Copper, Aluminium, Nickel^22^, and Platinum have been advanced for plasmonic applications^23^. Copper nanoparticles, due to their cost-effective and abundant properties along with the strong broadband absorption range (visible to near-infrared) have been interesting as a plasmonic non-noble metal in various fields^24^ including solar-based water desalination^25–27^. For example, copper oxide in an aerogel structure composed of polyvinyl alcohol and chitosan was used as a plasmonic SSG system with a solar-thermal conversion efficiency of 87.1%^28^. A composite light absorber includes hollow structures of CuS and a cellulose film was fabricated by solvothermal method to achieve an energy conversion efficiency of 85%^29^. Ali et al. proposed an in-situ solvothermal method to load the 3-D hierarchical Cu_3_SnS_4_ on the wood substrate to introduce a high steam generation efficiency of 90%^30^. In addition, a copper sulfide-based solar evaporator through a hydrothermal method records a high water evaporation efficiency of ~ 90%^31^. CuS in a polymeric hybrid membrane with polyethylene also showed effective full-spectra solar absorption properties with a conversion efficiency of ~ 64%^32^. To the best of our knowledge, there are few reports on plasmonic-based solar evaporators based on flexible textile^33^ as a substrate some of them have been briefly mentioned as follows: (1) a functionalized cotton pad with copper sulfide nanocage in an aerogel structure was considered a flexible photothermal absorber with a high energy conversion efficiency of ~ 95%^34^. (2) plasmonic cotton fabric with gold and silver nanoparticles was introduced as a flexible and low-cost photothermal light-absorber with a solar-thermal efficiency of up to 86%^35^. (3) a copper-sputtered layer on a leaf has been fabricated to develop a solar evaporator with water transmission through the cotton and a vaporization efficiency of ~ 84%^36^.

Fast oxidation of Cu nanoparticles makes them unstable under atmospheric conditions which hinders their applications^37^. So, several strategies have been implemented to enhance their stability such as complex structures e.g. core/shell copper nanoparticles and copper oxide-based systems^38^. Furthermore, the toxicity of chemicals used in synthesized nanoparticles led to emergent concerns for researchers. Developing reducing agents with plant resources^39, 40^ is being as an engaged and environmental-friendly approach owing to the green synthesis of metallic nanoparticles^41^. *Pterocarpus santalinus,* also known as red sandalwood, or red sanders, contains many compounds with medicinal properties in cure diabetes^42^, bilious infections, and skin diseases^43^. Some of the components of red sanders’ aqueous extraction are flavonoids, glycosides, tannins, sterols, saponins, phenols, and alkaloids^44^. The flavonoids and sugars were reported as the main contributors to reducing the metal salt solutions to the nano state^45^. Proteins, glucose, and some others are responsible for capping the generated particles^46^ along with descending their agglomerations^40^. Indeed, diverse studies indicate that the red sanders’ callus exhibit antibacterial features against common human pathogenic bacteria, fungi, and protozoa with anti-oxidant characteristics^43^.

In the present work, the aqueous extraction of red sanders was used for the in-situ generation of durable copper nanoparticles on the cotton fabric. Copper-coated fabric, polyurethane foam, and the cotton yarn stitched on the foam were applied as the photothermal light-absorber, self-floating insulator, and in our plasmonic solar steam generation system, correspondingly. Simple, scalable, green, and stable synthesis of the Cu nanoparticles on t washable and flexible cotton substrate with anti-bacterial activities indicates the potentiality of our solar-based evaporators with a solar-thermal conversion efficiency of ~ 98%.

## Results

### Fabric characterization

**Figure 1:**
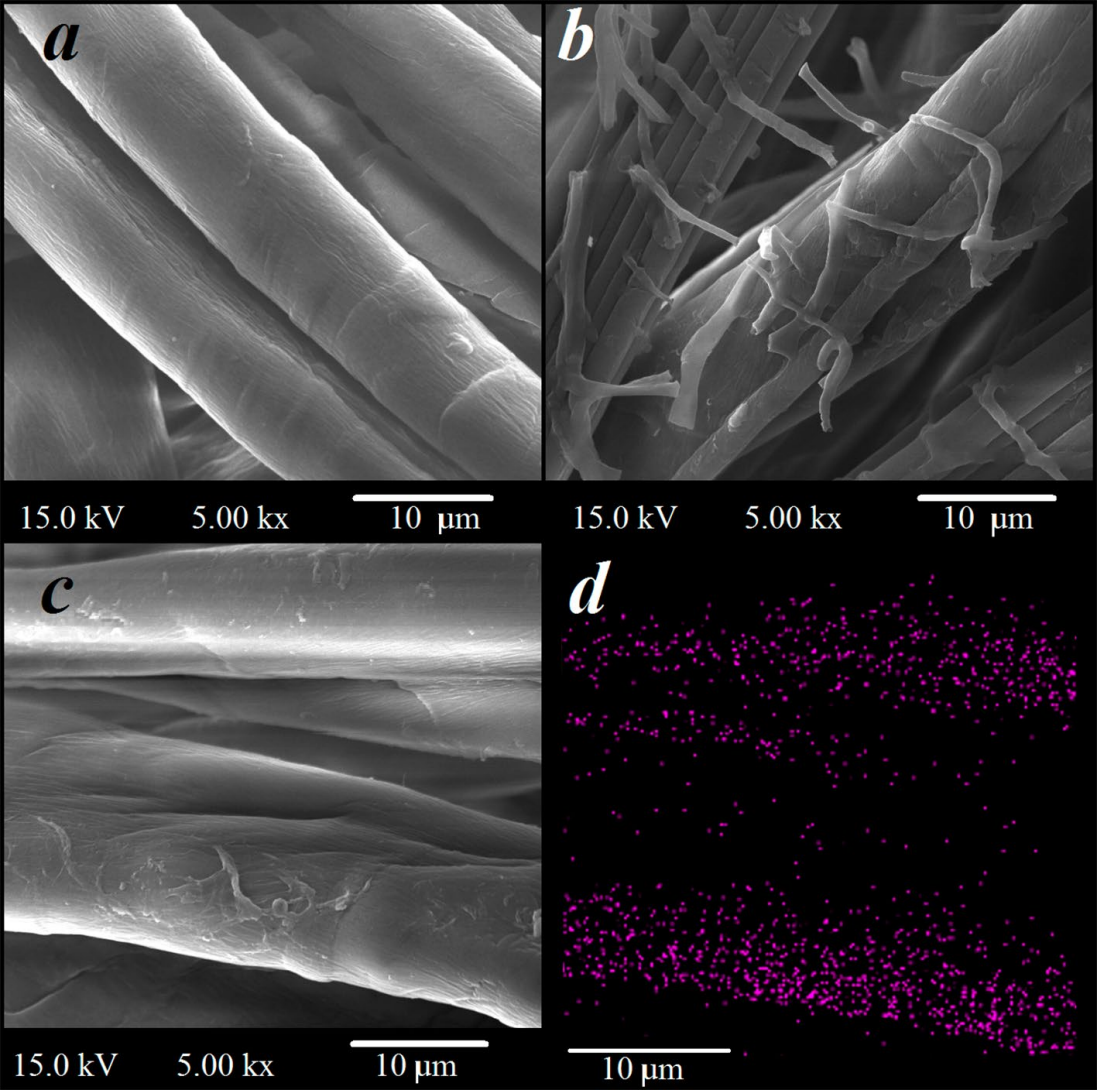
FESEM images of the pristine (**a**) and treated fabrics with red sanders (**b**). FESEM and EDS mapping of the Cu-treated fabric (**c**, **d**).

To describe the detailed structure of the pristine and treated fabrics with red sanders and Cu nanoparticles FESEM imaging (Fig. 1a–d) was carried out. The coating of the red sanders (matrix) with the fibral structure on the cotton fabric is shown in Fig. 1b. Due to the in-situ generation of Cu nanoparticles on the matrix substrate, the observation of nanoparticles is not easy (Fig. 1c). Therefore, the presence of Cu nanoparticles was confirmed by EDS mapping (Fig. 1d).

**Figure 2:**
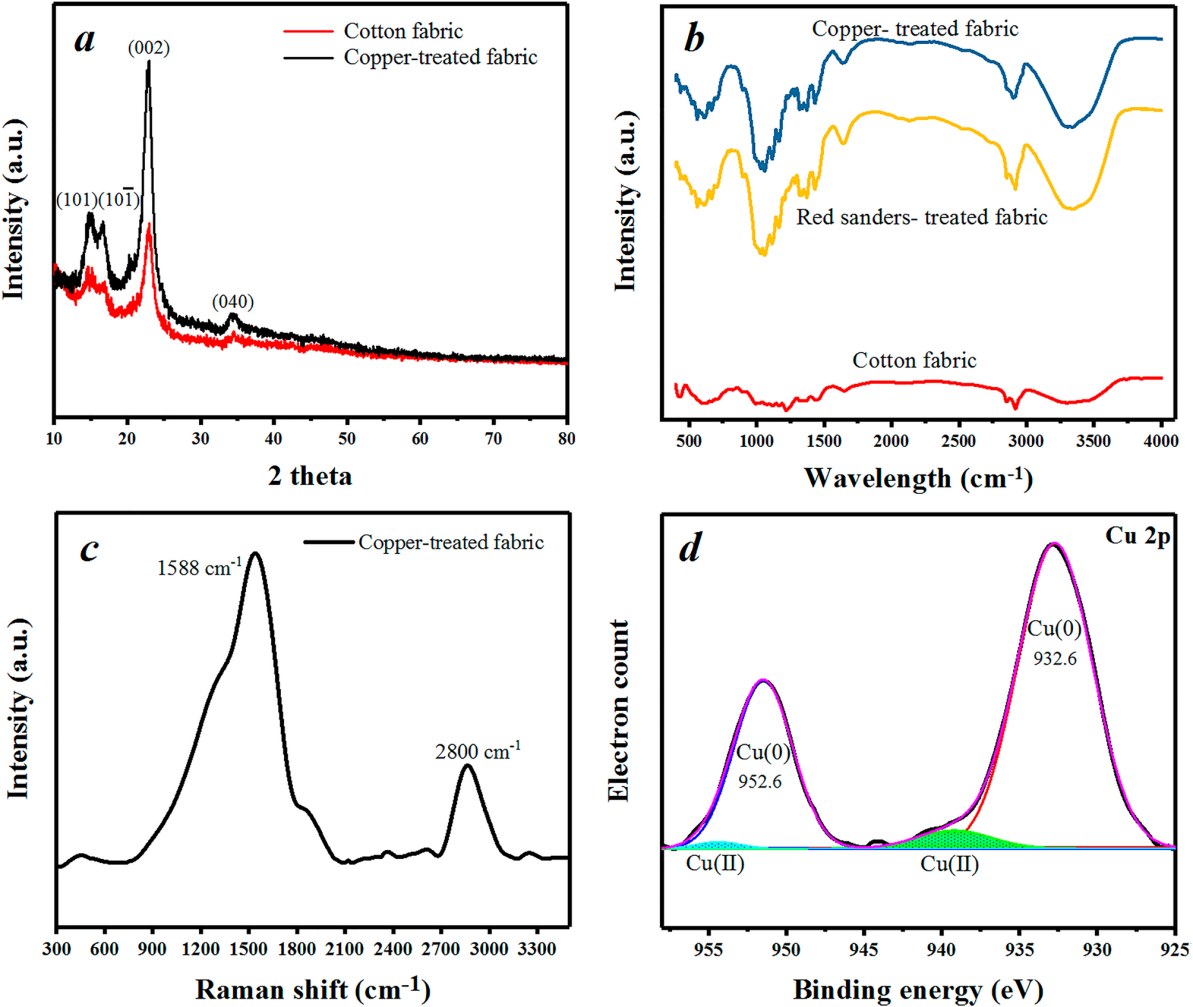
XRD (**a**), FTIR (**b**), Raman (**c**), and XPS (**d**) patterns of the fabrics.

The crystalline structure of the pristine and copper-treated fabric was revealed with X-ray diffraction patterns as shown in Fig. 2a. Microcrystalline cellulose structure of the cotton fabric shows four characteristic peaks at 14.6°, 16.5°, 22.7°, and 34.4° corresponded to (101), (101), (002), and (040) planes^47^. Because of the penetration depth of X-ray in XRD analysis, the spectrum presented no change after copper treatment^48^, indicating the stability of the fabric structure. To clarify the coating FTIR and Raman analyses have been utilized. The FTIR results of the pristine and treated fabrics are shown in Fig. 2b. In the treated samples, the peak at 1200–1250 cm^−1^ disappeared owing to OH deformation^49^. The C=O group formation was introduced with a new weak peak at around 1450–1500 cm^−1^ corresponding to cellulose oxidation due to the reduction process of copper ions^50^. In addition, OH bond stretching vibration at 3200–3500 cm^−1^ is observed with a significant increase in the red sander-treated sample. Therefore, the chemical absorption of the Cu nanoparticles besides the physical attachment can be revealed^44^. Raman spectroscopy results of the copper-treated fabrics is shown in Fig. 2c. The strong peak at 1588 cm^−1^ is assigned to non-oxidant copper species^51^ with no significant peak in the range of 300–600 cm^−1^^52^ indicating a zerovalent chemical state of the copper nanoparticles. The overlapping peak at the center of 2800 cm^−1^^53^ is characteristic of the cellulose structure in the treated sample. To investigate the chemical state of the Cu in the copper-treated fabric the XPS spectra were carried (Fig. 2d). XPS signals of Cu 2p result in sharp peaks at 952.6 and 932.6 eV binding energies corresponding to the zero oxidation state of copper. Two weak peaks at about 957.5 and 937.6 eV (colored-fill region) are ascribed to the slight surface oxidation (4.95%) of copper due to sample storage conditions at atmospheric conditions.

### Solar evaporation performance

**Figure 3:**
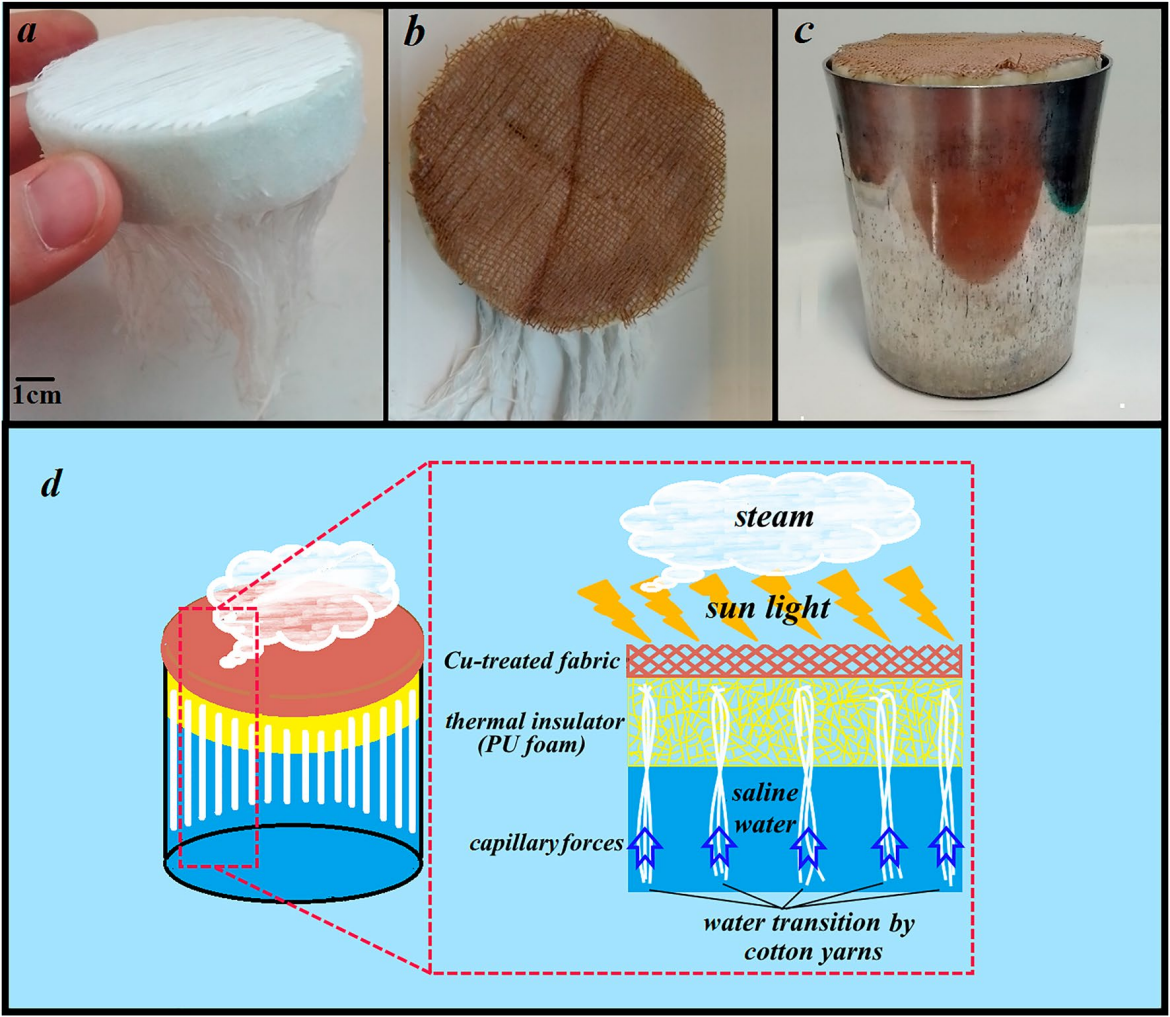
(**a**) The cotton yarn-stitched foam, (**b**) the treated fabric on the foam, (**c**) the proposed solar steam generator containing the foam, absorber, and water, and (**d**) the schematic of the SSG compositions.

As previously reported, a homemade solar simulator with a temperature controller of ± 1 °C was used^54^. It includes four water containers with the equal and uniform illumination power of one sun. Each container consists of the following parts: (1) A compressed PU foam, as the self-floating insulator that was stitched with cotton yarn (inspired by the jellyfish structure) to support continuous water transmission through capillary force from the container to the foam surface. This structure was used as the control sample. (2) The treated fabric samples as light absorbers were placed on the test holder position (Fig. 3).

**Figure 4:**
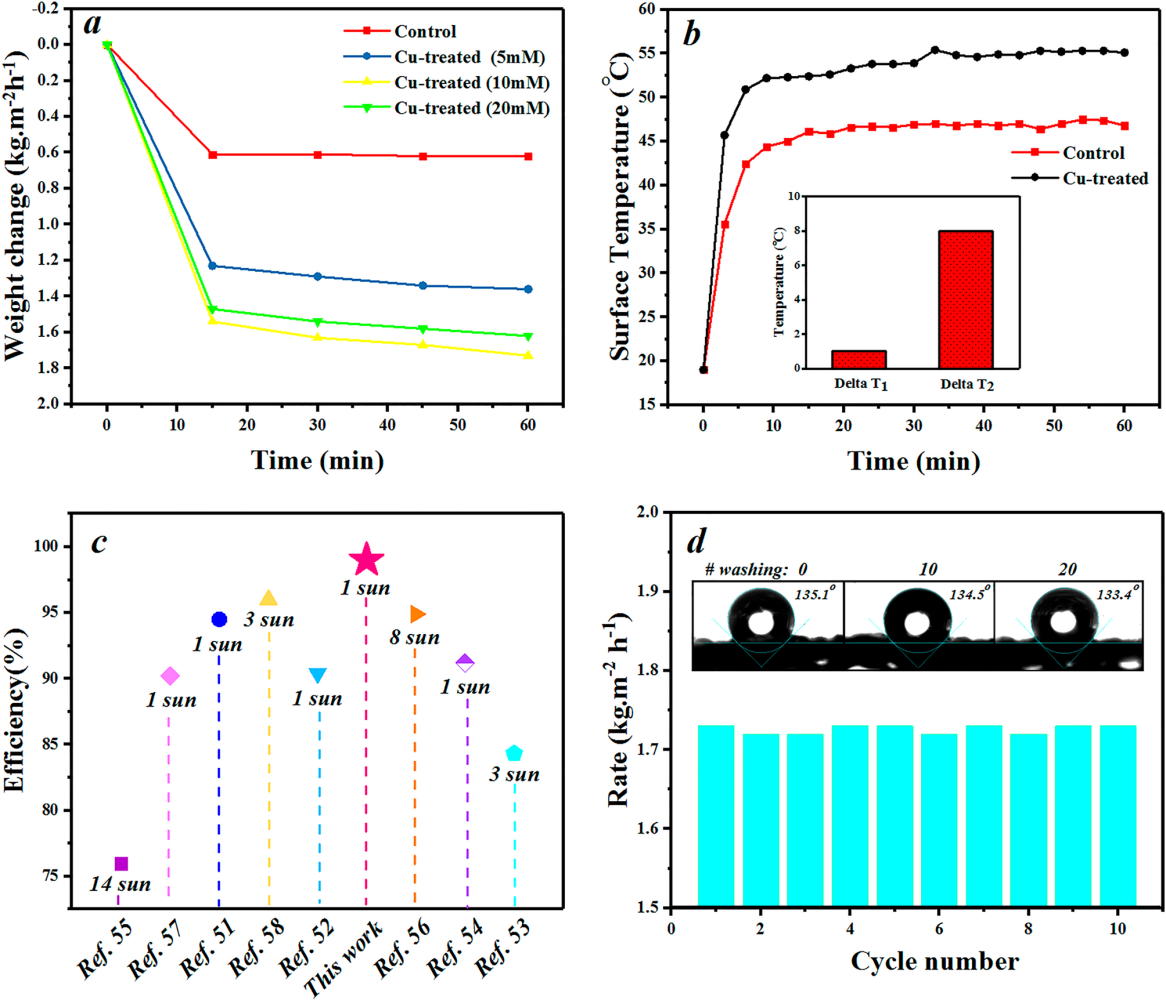
(**a**) Weight loss of the control and copper-treated fabrics under illumination of 1 kW m^−2^. (**b**) Surface temperature as a function of time for the control and test samples. The inset shows the ΔT_1_ and ΔT_2_ during solar irradiations. (**c**) Efficiency comparison of this work with some plasmonic SSG reports^56–63^. (**d**) Recyclability performance of the solar-based evaporator with a photograph of washing stability for fabric coating. The inset shows the water contact angles of the copper-treated fabric before and after washing.

The performance of the treated fabric was monitored by recording the weight loss of the water container every 15 min under irradiation (Fig. 4a). To evaluate evaporation rates, the slope of collected data was extracted and measured as: 0.62, 1.36, 1.73, and 1.62 kg m^−2^ h^−1^ for the control, and other treated fabrics with the initial concentration of the copper sulfate solutions of 5, 10, and 20 mM, respectively. The maximum evaporation rate was achieved for copper-coated fabric with a concentration of 10 mM which is 2.79 times higher than the control one. It seems that the reducing ability of the matrix is not enough for more concentrations than 10 mM. Therefore, this sample was selected as the optimum sample for the next characterizations and measurements.

The temperature variations of two points, including 1 cm from the bottom of the container (T_1_) and the fabric surface (T_2_), were recorded during illumination (Fig. 4b). These two temperatures indicate the thermal insulation property of the compressed PU foam along with the photothermal property of the treated fabric. The data (Fig. 4b) display a rather saturation behavior for the surface temperatures after ~ 10 min of illumination with a maximum value of 55.4 °C for the copper-coated sample. The difference in T_1_ and T_2_, as important evaporation characteristics for the test sample compared to the control one, were obtained ~ 1 and ~ 8 °C, respectively. This represents a remarkably higher photon absorption property while the evaporation along with continuous water transmission (jellyfish-like structure) prevents further increase of the surface temperature. The low ΔT_1_ reflects feasible heat management of the foam.

## Discussion

The evaporation efficiency (η) as an important parameter describes the overall solar-thermal energy conversion which can be expressed as:

1 $$\eta = \dot{m}h/q$$

where $\dot{m}$, h and q are evaporation rate (kg m^−2^ h^−1^), a total of sensible heat (ignored in our calculations), evaporation enthalpy (kJ kg^−1^), and the light source energy (kW m^−2^), respectively^55^. The dark evaporation rate (0.16 kg m^−2^ h^−1^) was subtracted from the measured values. The evaporation enhancement and solar to thermal conversion efficiency, during 1 sun illumination over 60 min, were ~ 179% and 98%, respectively.

To have an evaluation study, solar-to-thermal conversion efficiency values from other reports on plasmonic solar-based evaporators were listed in comparison with the present work. According to Fig. 4c, the current system has a high light-to-heat conversion efficiency.

To study the sustainability of the copper-treated fabric, the cyclic evaporation repetition was carried out 10 times which was including 60 min of irradiation along with the washing process. A rather constant rate verifies robust coating stability against washing processes (Fig. 4d). In fact, a clear solution at the end of the sonication process of the samples in DI-water for 15 min confirmed the proper stability of the coating.

In addition, the ICP results of Cu concentration after the fabric immersion for 24 h was about 0.017 mg l^−1^ which verified the rather good stability of the evaporator. Also, static contact angle analysis with saline water (NaCl, 3.5 wt%) displays rather stable hydrophobic behavior with an average contact angle of 134.4° as is shown in the inset of Fig. 4d (before and after 10 and 20 times washing).

**Figure 5:**
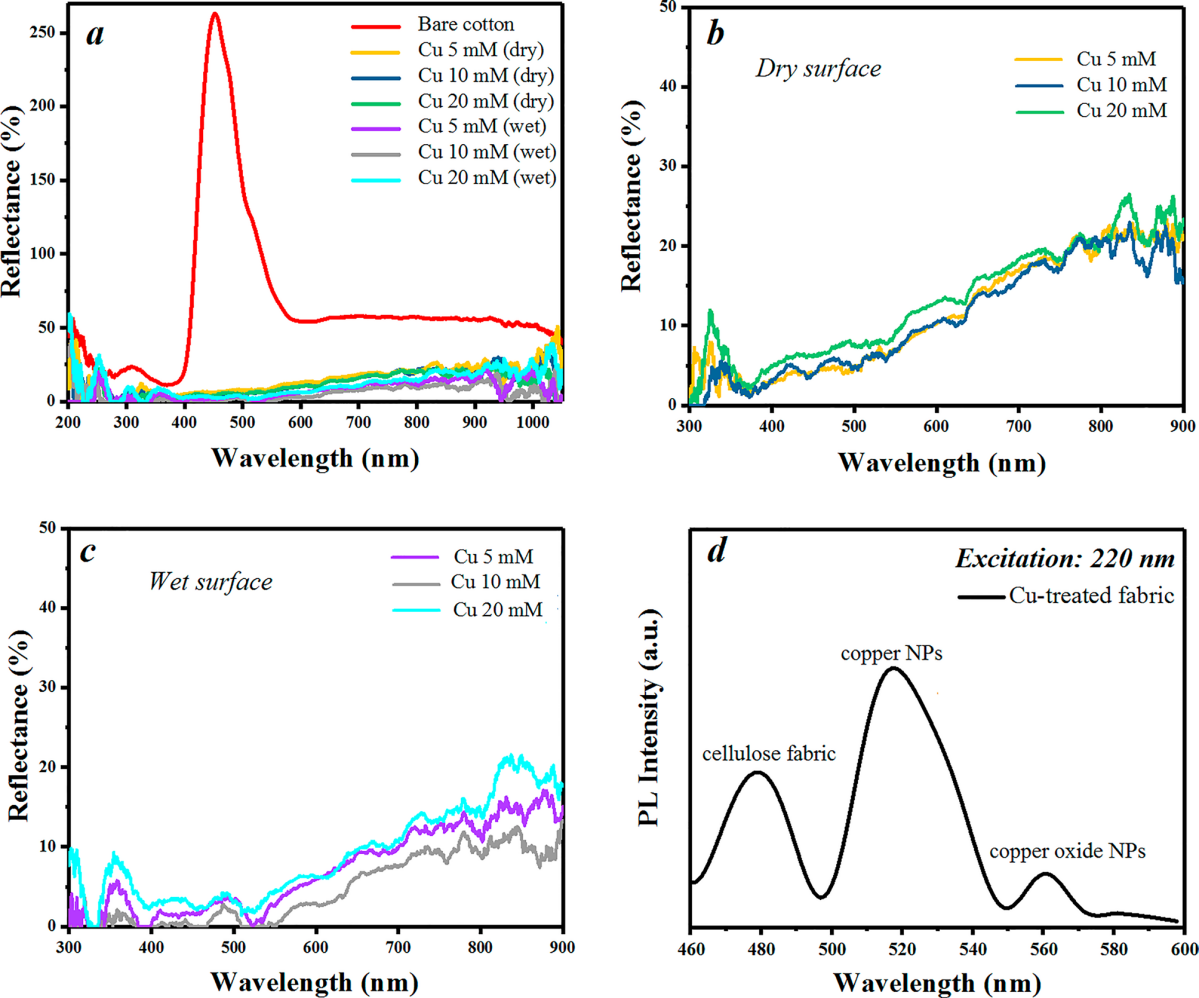
DRS curves of the bare and copper-treated fabrics in dry and wet (saline water) conditions (**a**). The zoomed DRS curves are shown in (**b**) dry and (**c**) wet conditions. Photoluminescence intensity of the Cu-coated fabric (**d**).

DRS analysis was employed to investigate the light absorption properties of the treated fabrics in the range of 200–1100 nm. The results are in comparison to barium sulfate powder as the reference sample with complete reflection at the analyzed range. The measurements were performed on all samples in both dry and wet conditions. According to Fig. 5a, the reflectance behavior of the copper-treated fabrics shows a remarkable decrease compared to pristine cotton. The sharp peak of an untreated sample in the range of 400–460 nm corresponds to the photoluminescence response of the cellulose structure^64^. Data for the samples with different copper concentrations shows reflectance below 20, and 10% for dry and wet conditions, respectively (Fig. 5b,c). The optimum sample (Cu 10 mM) shows a reflectance of lower than 5% in the wide range of the DRS spectrum. According to the *Kubelka–Munk* theory^65^, the absorption amount of this fabric is almost 90% for wet conditions. These results are in good consistency with the presented evaporation rates of the samples.

Photoluminescence (PL) emission spectra of the bare and copper-treated fabrics at room temperature were collected using a spectrophotometer (Fig. 5d). The emission spectrum of the Cu-treated fabric (excitation in 220 nm) shows a strong peak in the range of 500–550 nm ascribed to electron transition from valence (3d) to conduction band (4sp) introducing fluorescence of Cu nanoparticles^66^. The weak peak after 550 nm is related to the surface oxidation of copper nanoparticles on the fabric^67^. PL emission spectrum also presents a peak below 500 nm (Fig. 5d) related to cellulose photoluminescence in a similar range of the DRS curve (Fig. 5a).

**Figure 6:**
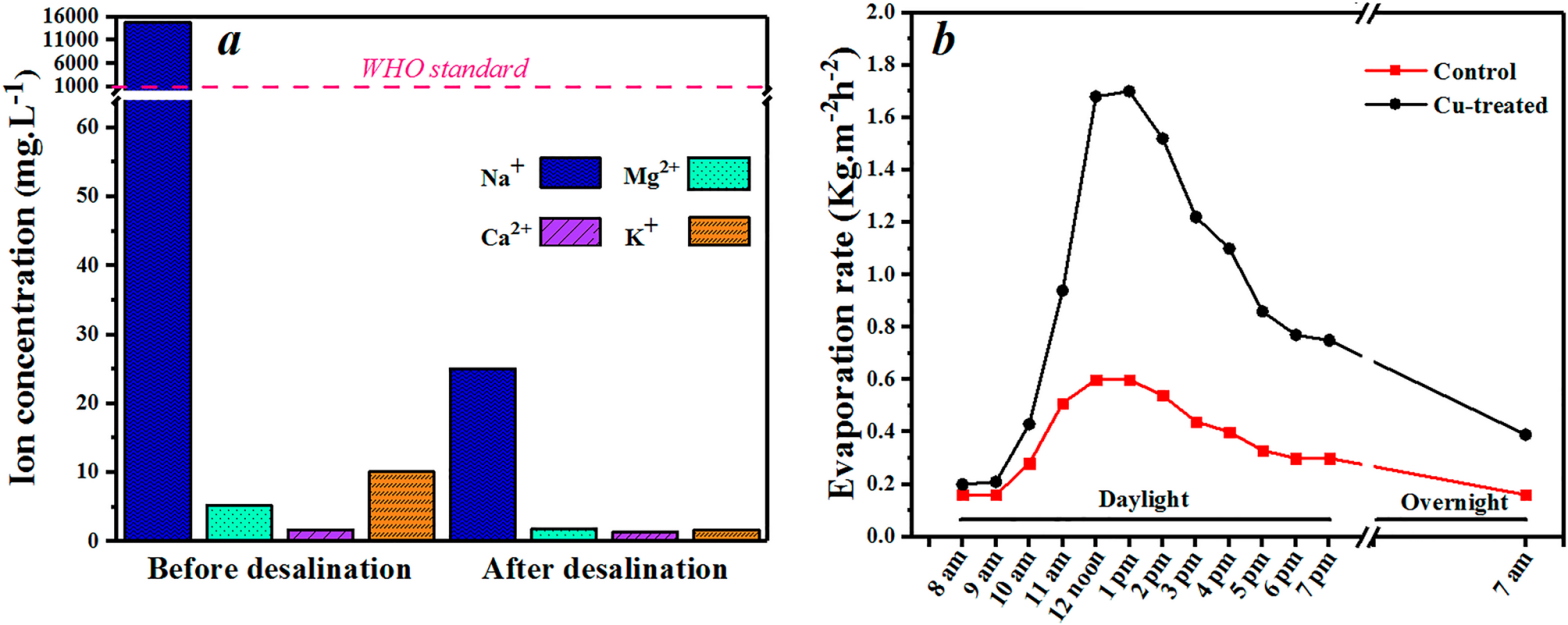
(**a**) Ion concentrations of Na^+^, Mg^2+^, Ca^2+^, and K^+^ after and before desalination. The dashed line shows the WHO standard for drinkable water^68, 69^. (**b**) Evaporation performance during a 1-day test under outdoor conditions.

The quality of the collected water from the prepared solar steam generator was studied by ICP analysis (Fig. 6a). The desalination performance was investigated by evaluation of some important ion concentrations (Na^+^, Mg^2+^, Ca^2+^, and K^+^) before and after illumination. The ion concentrations dropped impressively e.g. for sodium about 99.83%.

To study the performance of our solar-driven evaporator for practical applications, we measured the evaporation rates under outdoor conditions in daylight (12 h) and overnight (12 h), continuously (Fig. 6b). The results indicate an ongoing increase to a maximum rate of 1.68 kg m^−2^ h^−1^ at noon, a bit smaller than the indoor value (1.73 kg m^−2^ h^−1^) due to different ecological conditions.

**Figure 7:**
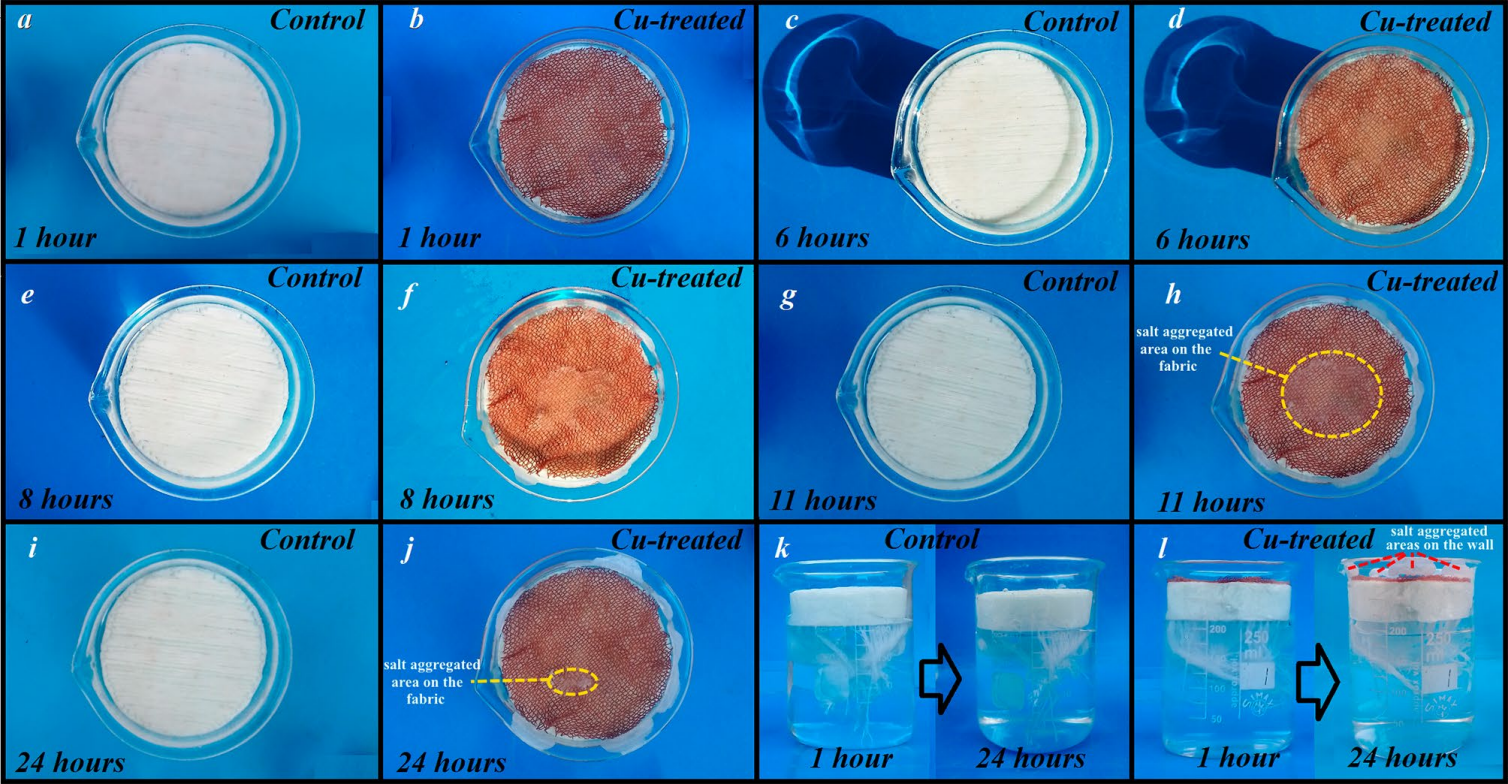
Photographs of the solar-based evaporator under outdoor conditions for 24 h.

One of the most important challenges of solar-based evaporators is salt aggregations. Salt crystallization leads to a gradual decline in the evaporation performance of the systems^70^. So, the antifouling properties of the prepared evaporator under outdoor conditions were investigated against salty water for 24 h (Fig. 7). The gradual salt aggregations were monitored only on the Cu-treated sample mostly along the walls due to relative hydrophilicity between fabric and glass container. During the night, salt on the fabric surface was reduced and on the glass wall increased simultaneously. This observation indicates a favorable effect for active surface regeneration in large-scale applications.

**Figure 8:**
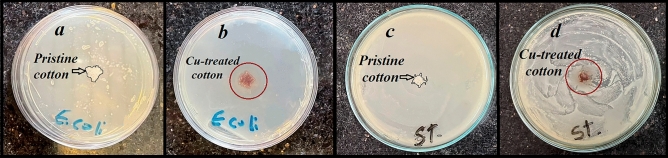
Antibacterial activity of the bare and copper-treated fabric against *E. coli* (**a**, **b**) and *S. aureus* (**c**, **d**).

Antibacterial activity of the copper-treated fabric against E. coli (gram-negative) and S. aureus (gram-positive) bacteria was investigated as side as useful properties of the treated samples. The disc diffusion test was employed to compare the clear zoon of the prepared fabric. According to Fig. 8a,c the pristine fabric with no observed clear zoon is unable to prevent bacteria growth around the fabric on the plate. While the copper-treated fabric showed an inhibition zoon diameter of ~ 18 (*E. coli*) and 15 (*S. aureus*) mm compared to the standard antibiotics as shown in Fig. 8b,d, respectively. These antibacterial activities can be considered desirable properties for a safe and efficient texture-based desalination system.

In this study, we used the plasmonic effect of the copper nanoparticles in an interfacial solar-based evaporator. In the prepared solar steam generation system, in-situ generation of nanoparticles was carried out by the red sanders matrix as a green reducing agent in the cotton fabric substrate. The treated fabric showed color stability for 1 year and also evaporation rate even after ten times illumination plus washing. It was set on the PU foam as a self-floating insulator that was stitched with cotton yarns to have a jellyfish structure. The system shows the evaporation rate and enhancement of 1.73 kg m^−2^ h^−1^, and ~ 179% with the appropriate self-recovery properties during dark conditions. The examined structure possesses a high solar-thermal conversion efficiency of ~ 98%. Desalination data based on ICP analysis and antibacterial properties of the treated fabric indicate the simple, cost-effective, scalable, and green preparation process for a highly efficient solar-based evaporator.

## Methods

### Material

White cotton fabric with a thickness of ~ 2 mm was provided by Hejab Company. *Pterocarpus santalinus* wood with the common name red sanders was purchased from the local market. Copper sulfate (Sigma-Aldrich Co., Ltd) and other chemicals were used in analytical grade, as received.

### Preparation of red sander extract

100 gr of red sander pieces with 900 mL of distilled water were stirred for 20 min at 80 °C. The extract was filtered and centrifuged (2000 rpm, 5 min) to get a clear red-colored solution.

### Preparation of the fabric with the reducent agent coating

The white cotton fabric was cleaned with acetone solution followed by washing with deionized water and dried at 60 °C. The pieces of the fabric (10 × 10 cm^2^) were dipped in 200 mL of the prepared extract under continuous stirring for 24 h at ambient temperature. The extract-coated fabrics, as the green reducent agent matrix for in-situ generation of copper nanoparticles, were rinsed with DI water and then dried at 60°C^44, 71^.

### In situ generation of copper nanoparticles on the reducer-coated fabric

**Figure 9:**
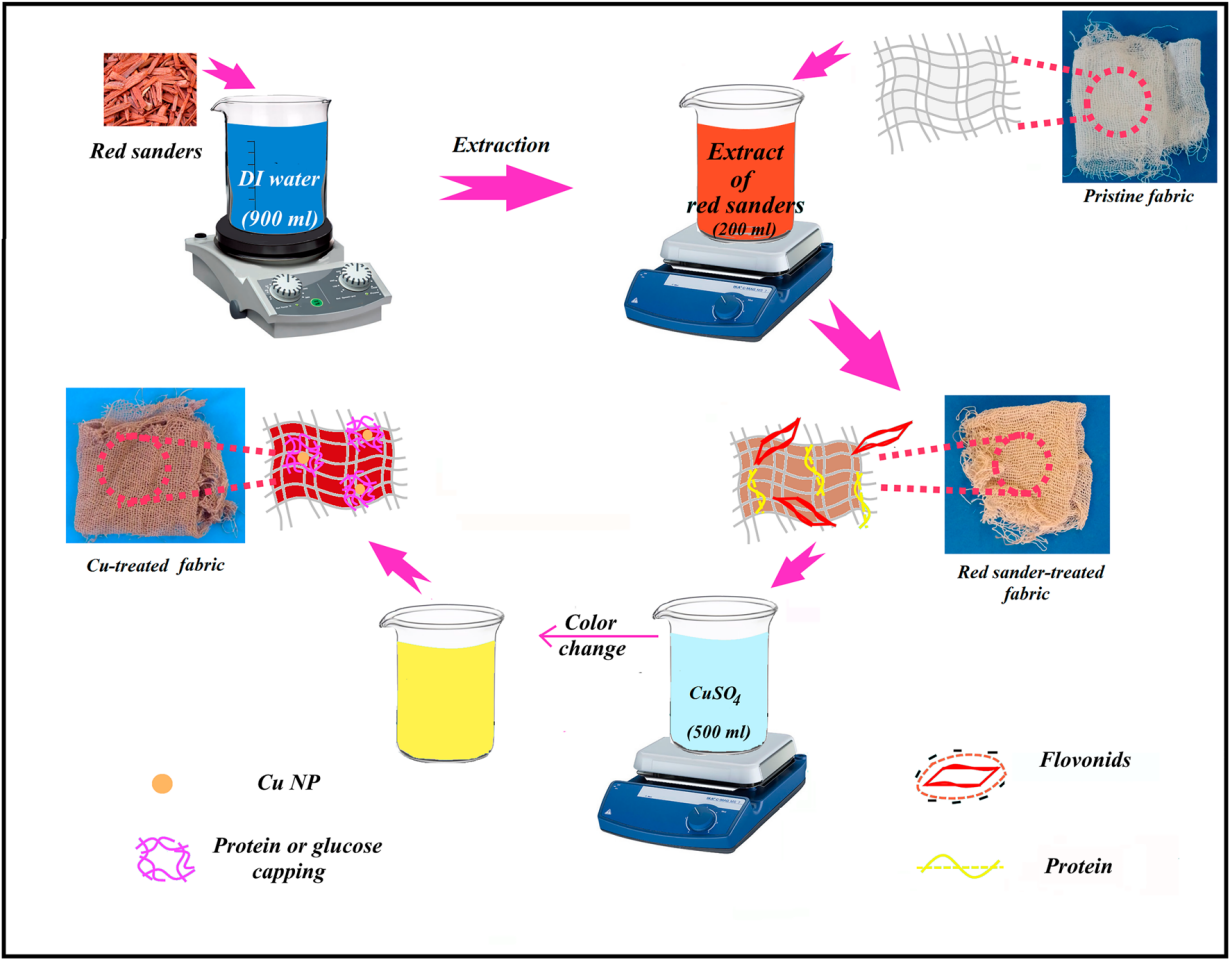
The schematic diagram of the red sanders extraction and in-situ generations of the Cu NPs on the cotton fabric.

The prepared matrix was dipped in the 500 mL aqueous solution of pentahydrated copper sulfate (CuSO_4_. 5H_2_O) with the specific concentrations (5, 10, 20 mM), and stirred for 24 h. After this, the fabric was washed and dried, respectively. The color change of the solution and matrix during stirring was the preliminary evidence for in situ generations of nanoparticles. Furthermore, the unchanged color after months shows the permanent formation of nanoparticles. The prepared fabric with an approximate thickness of 1 mm was used as the solar absorber. The digital photographs of the fabrics and the schematic of the synthesis process are shown in Fig. 9.

### Characterization

The morphologies of the matrix and copper NPs-coated fabrics were characterized by a field emission scanning electron microscope (FESEM, MIRA 3/TESCAN, Czech). X-ray diffraction (XRD, PANalytical B.V., EVISA, Netherlands) was carried out to study the crystal structure of the specimens (2θ: 10° to 80°, room temperature). Fourier transform infrared spectroscopy (FTIR) of the treated fabrics was analyzed in the frequency range of 4000–400 cm^−1^ by Tensor 27 FT-IR, Bruker. Raman spectra were acquired using HORIBA Scientific, Xplora PLUS confocal Raman spectrometer with 532 and 785 nm excitation lasers. The emission photoluminescence spectra of the fabrics were recorded at room temperature with a Cary Eclipse Fluorescence Spectrophotometer, Agilent Technologies. The excitation wavelength was 220 nm with the spectral range extending from 460 to 600 nm. Contact angle analysis was performed with Dataphysics OCA 15 plus device with a volume drop of 4.0 μl. The angle calculations and the photographic images were performed through the SCA 20 software and a CCD camera, respectively. The surface reflectance of the samples was investigated through differential reflectance spectroscopy (DRS), using Avaspec 2048 Tech coupled with Ava Sphere series integrating sphere and Avalight DHS lamp (200–1100 nm). The real-time temperatures of two points in the container and the surface of each sample were recorded by two typical digital temperature meters. Ion concentrations of the collected condensed water were studied by inductively coupled plasma optical emission spectrometry (ICP-OES, Spectro Arcos). Condensed vapor collection from the inner surface of a homemade glass was done as reported in previous work^54^. The water weight changes were measured by an electronic Notebook Pocket Scale mass balance (accuracy: 10^−5^ kg).

All methods were carried out in accordance with relevant guidelines.

## Data Availability

The datasets generated during and/or analysed during the current study are available from the corresponding author on reasonable request.
